# Molecular Pathomechanisms of Impaired Flow-Induced Constriction of Cerebral Arteries Following Traumatic Brain Injury: A Potential Impact on Cerebral Autoregulation

**DOI:** 10.3390/ijms22126624

**Published:** 2021-06-21

**Authors:** Annamaria Szenasi, Krisztina Amrein, Endre Czeiter, Nikolett Szarka, Peter Toth, Akos Koller

**Affiliations:** 1 Department of Translational Medicine, Faculty of Medicine, Semmelweis University, 1085 Budapest, Hungary; ancsi.szenasi@gmail.com; 2 Department of Morphology and Physiology, Faculty of Health Sciences, Semmelweis University, 1085 Budapest, Hungary; 3 Department of Neurosurgery and Szentágothai Research Center, University of Pecs, Medical School, 7623 Pecs, Hungary; tina.amrein84@gmail.com (K.A.); endre.czeiter@aok.pte.hu (E.C.); petitoth@gmail.com (P.T.); 4 MTA-PTE Clinical Neuroscience MR Research Group, 7623 Pecs, Hungary; nikiszarka@gmail.com; 5 Research Center for Sports Physiology, University of Physical Education, 1123 Budapest, Hungary; 6 Department of Physiology, New York Medical College, Valhalla, NY 10595, USA

**Keywords:** hemodynamic forces, diameter, autoregulation, arachidonic acid, CYP450 4A, TXA_2_ receptors

## Abstract

(1) Background: Traumatic brain injury (TBI) frequently occurs worldwide, resulting in high morbidity and mortality. Here, we hypothesized that TBI impairs an autoregulatory mechanism, namely the flow-induced constriction of isolated rat middle cerebral arteries (MCAs). (2) Methods: TBI was induced in anaesthetized rats by weight drop model, and then MCAs were isolated and transferred into a pressure-flow chamber. The internal diameter was measured by a video-microscopy. (3) Results: In MCAs from intact rats, increases in flow and pressure + flow elicited constrictions (−26 ± 1.9 µm and −52 ± 2.8 µm, *p* < 0.05), which were significantly reduced after TBI or in the presence of thromboxane-prostanoid (TP receptor) antagonist SQ 29,548. Flow-induced constrictions were significantly reduced by HET0016, inhibitor of cytochrome P450 4A (CYP450 4A). Arachidonic acid, (AA, 10^−7^ M), and CYP-450 4A metabolite 20-hydroxyeicosatetraenoic acid (20-HETE) elicited constrictions of intact MCA (−26 ± 2.3% and −31 ± 3.6%), which were significantly reduced after TBI (to 11 ± 1.3% and −16 ±2.5%). The TP receptor agonist U46619 (10^−7^ M) elicited substantial constrictions of MCA from intact rats (−21 ± 3.3%), which were also significantly reduced, after TBI (to −16 ± 2.4%). (4) Conclusions: Flow-induced constrictor response of MCA is impaired by traumatic brain injury, likely due to the reduced ability of cytochrome P450 4A to convert arachidonic acid to constrictor prostaglandins and the mitigated sensitivity of thromboxane-prostanoid receptors.

## 1. Introduction

Regulation of cerebral blood flow (CBF) is accomplished by several mechanisms acting on multiple levels. Some of them are serving local needs [1,2,3,4,5], whereas others ensure that an appropriate volume of the blood is flowing into the brain seated in the rigid cranium. These later ones are important because they can prevent increases intracranial pressure by preventing large amount of blood volume entering the brain [4]. These mechanisms are located in the vascular wall and sensing changes in hemodynamic forces related to pressure [6,7,8,9,10] and flow [4,11,12,13,14] and convert them into vasomotor responses eliciting thereby autoregulation of cerebral blood flow i.e., relatively constant flow in the face of changes in systemic blood pressure (in a range of: ~60–160 mmHg).

The presence of pressure sensitive myogenic response of cerebral arteries has been recognized early on [11,12,15,16], whereas in the last decade, the flow sensitive autoregulatory mechanism has been described [4,15,17]. It seems that both mechanisms are necessary to elicit autoregulation by providing together frank constrictions of cerebral arteries that sufficiently increase vascular resistance to prevent increases in blood flow when perfusion pressure increases [4].

Previous studies have shown that arachidonic acid (AA) metabolites are intimately involved in the translation of hemodynamic forces into vasomotor responses of cerebral arterial vessels [12,15,18,19,20]. These metabolites are a product of cytochrome P450 o-hydroxylases CYP450 4A, such as 20-hydroxyeico-satetraenoic acid (20-HETE), which are acting on thromboxane-prostanoid receptors (TP receptors). In addition, various prostaglandins such as PGE_2_ were also produced [21,22] and have important roles in the regulation of cerebrovascular resistance [4,23]. It has been shown that upon increasing systemic blood pressure or flow (for example, during exercise, Valsalva maneuver or hypertension), AA is released from cell membrane and then metabolized by CYP450 4A into 20-HETE, which elicits substantial constrictions of cerebral arterial vessels by acting on TP receptors [15,24], thereby limiting the increase in cerebral blood flow and thus providing an autoregulation of CBF.

One of the conditions when the importance of autoregulation became evident is after traumatic brain injury (TBI). Unfortunately, TBI occurs very frequently, several millions per year worldwide, followed by high rates of morbidity and mortality [25,26]. In addition to headache, hemorrhage, and cognitive decline, one of the visible hallmarks of TBI is the development of brain edema. This is due to the fluid leakage elicited by the high intraluminal pressure in the brain microvessels. In our previous study, we proposed that this is because the proximal larger arteries cannot constrict to increases in intraluminal pressure after TBI. Indeed, we have found that TBI impairs the myogenic response of isolated middle cerebral arteries [27]. The potential changes in the flow sensitive vasomotor response, however, has not yet been revealed. Because both hemodynamic forces are coupled to vascular resistance changes and thus contribute to autoregulation, we thought it would be important to elucidate whether or not flow sensitive responses are affected by TBI.

Based on the above, we hypothesized that TBI impairs flow sensitive vasomotor response of isolated middle cerebral arteries (MCAs) of the rat. We selected this species and this type of vessel in order to make our results comparable to our previous findings [27,28]. In the presence of constant intraluminal pressure, in this study, we changed the intraluminal flow and followed changes in the diameter of MCAs isolated from intact and TBI groups of rats. By use of pharmacological tools, we also aimed to assess the potential changes in molecular mechanisms mediating flow-induced vasomotor responses before and after TBI.

## 2. Materials and Methods

### 2.1. Animals

All procedures were approved by the Institutional Animal Care Use and Committee of University of Pecs, Medical School, and the National Scientific Ethical Committee on Animal Experimentation, Budapest Hungary in accordance with the ARRIVE guidelines. Wistar–Kyoto rats (male 300–350 g) were purchased from Charles River Laboratories (Wilmington, MA, USA) and were used for all experiments. The fad standard rat chow and free access to tap water.

### 2.2. Traumatic Brain Injury (TBI) 

Impact acceleration diffuse brain injury was induced by Marmarou’s well-characterized weight drop model [11,16,29,30]. In brief, under isoflurane (2%) anesthesia [27,31,32], the skull was exposed by a mild-line incision between the lambda and bregma and a steel disc was fixed with dental acrylic on the skull. A 450 g cylindrical weight from 1.5 m was dropped onto the disc, causing diffuse traumatic brain injury to the animals. Middle cerebral arteries were isolated from animals who survived 24 h after TBI.

### 2.3. Isolation of Rat Middle Cerebral Arteries and Use of Flow-Chamber to Obtain Vasomotor Responses

In brief, rats were anesthetized and decapitated, and the brains were removed from the intact and TBI groups and placed in Krebs solution (37.5 °C). Middle cerebral arteries (MCAs) were isolated from both sides of the brain of the animals (*n* = 77) [24]. After isolation, cerebral arteries were transferred into a custom-made pressure-flow chamber. Inflow and outflow pressures were controlled and measured by a pressure servo-control system (Living Systems Instrumentation, Burlington, and VE, USA). Perfusate flow was measured by a ball flow meter (Omega, Omega Engineering Inc., Stamford, CT, USA). The internal diameter was measured by videomicroscopy with a microangiometer (Texas A&M University System, College Station, TX 77840, USA). Changes in arterial diameter were continuously recorded digitally by PowerLab system (AD Instruments, Sydney, Australia) connected to a computer for later analysis. The size of glass pipettes used in this study was matched to both each other and the diameter of the vessels to achieve equal resistance [33]. In addition, inflow and outflow reservoirs and position of the chamber were built in a symmetrical manner, providing equal pressures or generating flow in the presence of constant pressure in the midsection of the vessels.

### 2.4. Myogenic Tone and Flow-Induced Responses of Isolated Cerebral Arteries

By the end of the 60 min incubation, the vessels developed a spontaneous myogenic tone in response to 80 mm Hg of intraluminal pressure. Then, changes in diameter of MCA were obtained to stepwise increases in flow elicited by pressure differences (ΔP; established by changing the inflow and outflow pressure to an equal degree, but opposite direction. In previous studies [33], we measured flow corresponding to pressure difference: ΔP = 0, 10, 20, 30, and 40, which corresponded to 3 to 32 μL/min changes in intraluminal flow (at 80 mm Hg intraluminal pressure) [19]. The following general protocol was used: vessels were exposed to 3 min at each flow rate to reach a steady-state diameter. Flow–diameter relationships were obtained in various conditions in control and TBI groups. Drugs and inhibitors were administered into the vessels chamber.

### 2.5. Administration of Vasoactive Agents

Diameter responses of MCA were obtained in response to arachidonic acid (AA) (10^−7^, and 10^−8^ mol/L), and cytochrome P450 o-hydroxylases (CYP450 4A) metabolite 20-hydroxyeicosatetraenoic acid (20-HETE, 10^−7^ mol/L) and 9,11-Dideoxy-11α, 9α-epoxymethanoprostaglandin F2α (U46619, 10^−6^; 10^−7^ mol/L), a synthetic analog of the PGH_2_ acting on the thromboxane-prostanoid (TP) receptors, as an agonist in various conditions.

Administration of Inhibitors: Diameter responses of MCA were obtained in the absence and presence of N-(4-butyl-2-methylphenyl)-N’-hydroxy-methanimidamide (HET0016, 10^−6^ mol/L an inhibitor of CYP450 4A producing 20-HETE [12] for 30 min to elucidate its role.

Additionally, we used [1S-[1α,2α(Z),3α,4α]]-7-[3-[[2-[(phenylamino)carbonyl]hydrazino]methyl]-7-oxabicyclo[2.2.1]hept-2-yl]-5-heptenoic acid (SQ 29,548; 10^−6 ^mol/L) aTP receptor blocker for 30 min to elucidate the role of this receptor in the vasomotor responses of MCA.

At the end of the experiments, the passive diameters were measured in the presence of Ca^2+-^free physiological salt solution (PSS) containing dimethyl 2,6-dimethyl-4-(2-nitrophenyl)-1,4-dihydropyridine-3,5-dicarboxylate (nifedipine 10^−5^ mol/L).

All drugs were purchased from Sigma Aldrich (St Louis, MO, USA), except SQ 29,548 and HET0016 (Cayman Chemical Company, Ann Arbor, MI, USA).

### 2.6. Quantitative Real-Time qRT-PCR

To analyze mRNA expression of the 20-HETE producing cytochrome isoforms CYP4a1 and CYP4a3 in MCAs of rats from each group, we used a quantitative real-time qRT-PCR technique as previously [34]. According to the protocol suggested by the manufacturer (Life Sciences, Carlsbad, CA, USA), total RNA was isolated with the Pure Link TM RNA Mini Kit. After sample homogenization, RNA was purified by ethanol treatment and eluted from the membrane. Using NanoDrop (Thermo Scientific, Waltham, MA, USA), the total amount of RNA was determined. Then, the high capacity cDNA kit was applied to perform cDNA synthesis (Applied Biosystems, Foster City, CA, USA). qRT-PCR was performed using SensiFast SYBR Green reagent (BioLine, Luckenwalde, Germany) for gene expression analysis. Amplifications were run on ABI StepOnePlus system (Applied Biosystems, Foster City, CA, USA). StepOne software was used to analyze gene expressions, which was normalized to the 18S housekeeping gene. Based on the quality of the PCR reference curves, the 18S was chosen as a reference gene. The primer sequences were: 18S: forward: TTGCTGATCCACATCTGCTGG, reverse: ATTGCCGACAGGATGCAGAA; CYP4a1: forward: AATGCTAACCCCAGCCTTCC, reverse: AGAGGAGTCTTGACCTGCCA; CYP4a3: forward: CTGTAGCTTTTCCTCCAGACTCCA, reverse: CAGTGGCTGGTCAGAGGTGAA. The amplification of PCR products was calculated according to the 2^−ΔΔCt^ method.

### 2.7. Statistical Analysis 

Statistical analysis was performed by two-way ANOVA followed by a Tukey’s post hoc test or Student’s *t*-test. All data are presented as mean ± SEM and *p* values < 0.05 were considered significant. Data are reported either in micrometer (µm) or as percentage of passive diameter at the corresponding intraluminal pressure.

## 3. Results

Flow-Induced Responses of Cerebral Arteries and Traumatic Brain Injury

The active, pressure-induced diameter of MCAs isolated from intact rats was 238.2 ± 6 µm, whereas the passive diameter was 280.9 ± 9 µm (*n* = 16, *p* < 0.05). Thus, in the presence of 80 mmHg intraluminal pressure, a substantial myogenic tone developed in MCAs.

**Figure 1:**
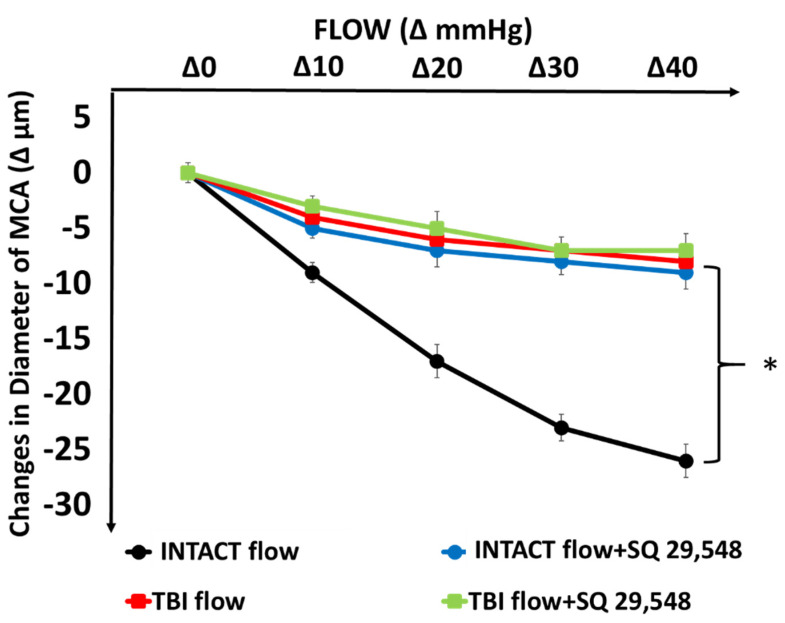
Changes in diameter of isolated middle cerebral arteries (MCAs) in response to step increases in intraluminal, flow (Δ0, Δ10, Δ20, Δ30, Δ40 mmHg between inflow and outflow cannulas) from intact rats and from rats after traumatic brain injury (TBI) in the absence and presence of SQ 29,548 (10^−6^ mol/L) an inhibitor of thromboxane A_2_ receptor (TP). Asterisks indicate significant differences * *p* < 0.05 vs. Intact flow and Intact flow with SQ 29,548 (*n* = 16, *n* = 8 in each group).

In intact MCAs increases in flow (by increasing pressure difference, in the presence of constant pressure of 80 mmHg) elicited substantial constrictions of diameter (Δ −26 ± 4 µm, *n* = 16, *p* < 0.05) as shown in Figure 1. After traumatic brain injury (TBI), constrictions to increases in flow were greatly and significantly reduced (Figure 1).

Similarly, in the presence of SQ 29,548 (10^−6^ mol/L), an inhibitor of thromboxane-prostanoid receptor (TP receptors) flow-induced constrictions of MCA from intact rats were significantly reduced and after TBI, the reduced constrictor responses were not further affected by SQ 29,548.

SQ 29,548 compound is a specific inhibitor of TP receptors, thus the finding that in the presence of SQ 29,548, flow-induced constriction was inhibited indicates that TP receptors are involved in the flow-induced response confirming our previous findings [28]. This is shown in Figure 1, namely that in intact MCA, inhibition of TP receptors by SQ 29,548 significantly reduced flow-induced constrictions. The new finding of the present study is that after TBI, the reduced flow-induced constriction was not further affected by SQ 29,548, suggesting that this signaling pathway is impaired by TBI. 

**Figure 2:**
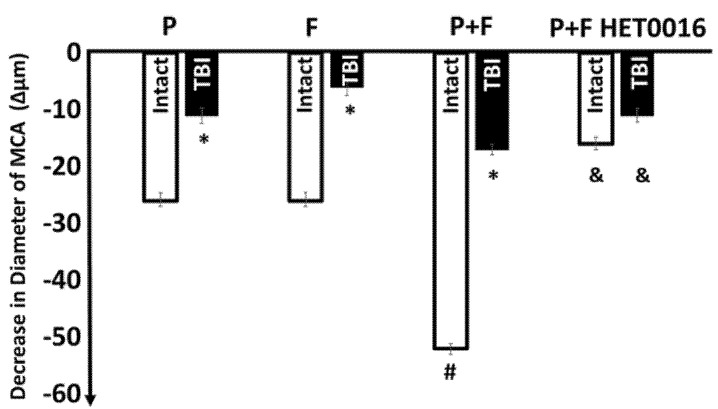
Shows the pressure (P)- or flow (F)-induced decreases in diameter alone, or the additive effect of pressure + flow on the diameter of MCA from intact rats. Additionally, Figure 2 shows that traumatic brain injury (TBI) significantly reduced the pressure (P)-, flow (F)-, and thus combined pressure + flow (P+F)-induced constrictions of MCAs (* indicates significant changes from Intact, # from P alone and F alone, and & from P+F (*n* = 24, data are mean ± SEM, *p* < 0.05) Moreover, this figure shows that HET0016 (10^−6^ mol/L), an inhibitor of cytochrome P450 4A (CYP450 4A), inhibited substantially and significantly the constriction induced by pressure + flow (*n* = 6 in each group).

To demonstrate the effects of hemodynamic forces on the maximum decreases in diameter of MCA in intact condition and after TBI, we plotted the data in a bar diagram (Figure 2). This figure shows the effect of pressure alone, flow alone, and pressure + flow together on the diameter of isolated MCA. It can be seen that together these hemodynamic forces elicited substantial and significant constrictions of MCA (Δ −52 ± 2.8 µm). Additionally, that HET0016, an inhibitor of CYP450 4A significantly inhibited diameter responses.

**Figure 3:**
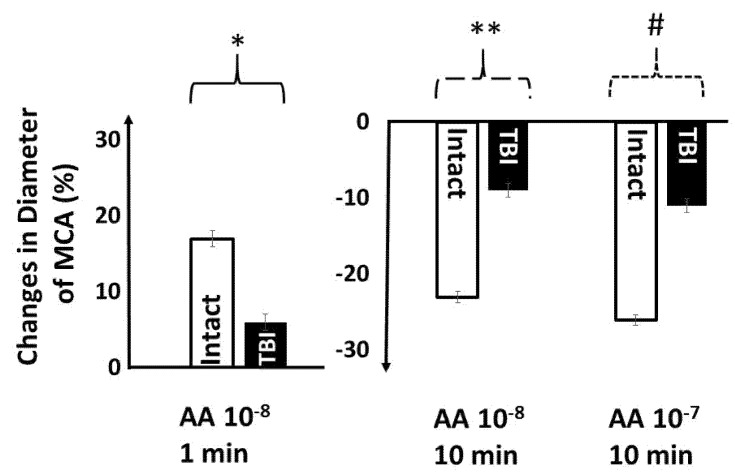
In middle cerebral arteries (MCAs) isolated from intact rats, lower concentration of arachidonic acid (AA), after initial dilations, (**left** panel), elicited constrictions (**right** panel), whereas higher concentration of AA elicited only constriction. After traumatic brain injury (TBI), AA-induced responses were significantly reduced. Data are mean ± SEM of normalized diameter %. (*, **, # indicate *p* < 0.05).

In order to prove that TBI interferes with the production of arachidonic acid (AA) metabolites and thus vasomotor responses, we tested AA-induced changes in diameter of MCA in various conditions (Figure 3). We found that in intact MCAs AA 10^−8^ mol/L, after initial dilation (at 1 min: 17 ± 2.1%), elicited constriction (at 10 min −23 ± 1.8%), whereas AA 10^−7^ mol/L at 10 min elicited constriction (−26 ± 2.3%). Importantly, AA-induced responses were significantly reduced in MCA isolated from the TBI group: AA (10^−8^ mol/L at 1 min: 6 ± 1.4%), after 10 min (10^−8^ mol/L: −9 ± 1.1%) and (10^−7^ mol/L: −11 ± 1.3%).

**Figure 4:**
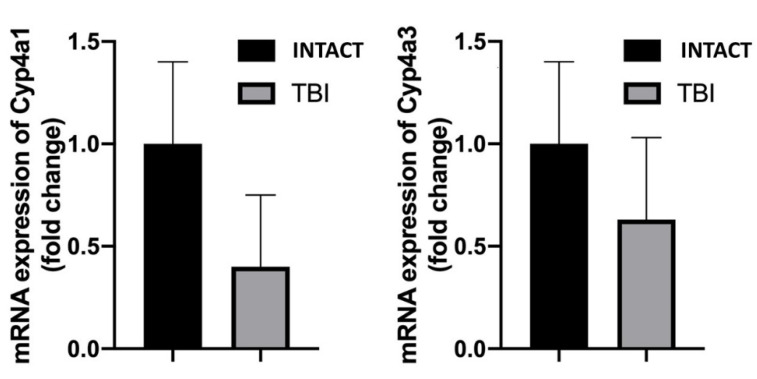
This figure shows the mRNA expressions of the cytochrome isoforms Cyp4a1 and Cyp4a3 in middle cerebral arteries (MCAs) of INTACT and TBI rats, which were substantially reduced after TBI (*n* = 6 in each group).

Next, we aimed to assess the mRNA expression of the 20-HETE producing cytochrome isoforms CYP4a1 and CYP4a3 in MCAs of rats from each group by using a quantitative real-time qRT-PCR technique as previously [34]. We found substantial reductions in the mRNA expressions of both CYP4a isoforms, corresponding to reduce vasomotor responses (Figure 4).

Next, we investigated the sensitivity of TP receptors to agonists. Early studies have shown that in cerebral arterial vessels, 20-hydroxyeicosatetraenoic acid (20-HETE), metabolite of cytochrome P450 o-hydroxylases (CYP450 4A), elicit constriction acting on thromboxane-prostanoid receptors (TP receptors) [15,20]. 

**Figure 5:**
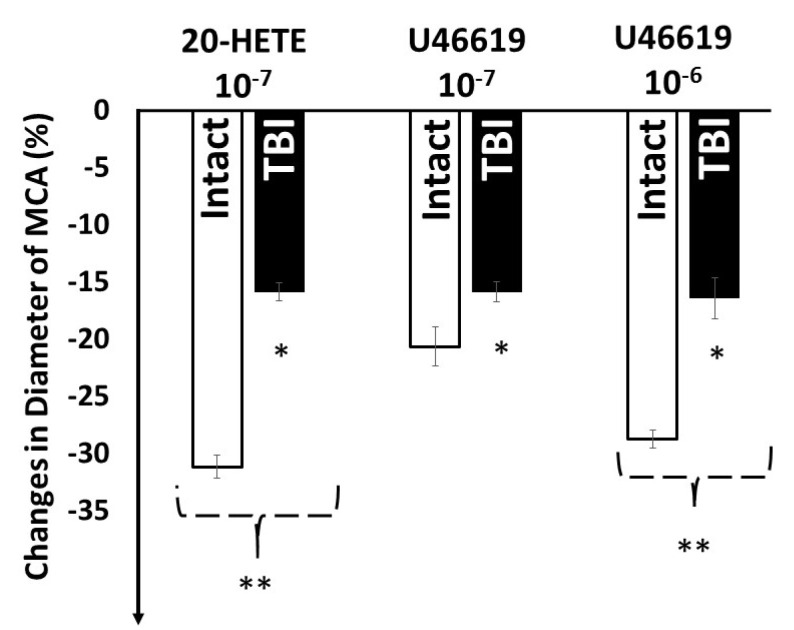
Changes in diameter of isolated middle cerebral arteries (MCAs) in response to 20-hydroxyeicosatetraenoic acid (20-HETE, 10^−7^ mol/L) metabolite of cytochrome P450 4A enzyme (CYP450 4A) and U46619 (10^−7^ mol/L and 10^−6^ mol/L a stable agonist of TP receptors, intact and after traumatic brain injury (TBI). Data are mean ± SEM. Asterisks indicate significant differences (* *p* < 0.05 from zero, Intact 20-HETE 10^−7^ ** *p* < 0.05 vs. TBI 20-HETE 10^−7^ and Intact U46619 10^−7^, Intact U46619 10^−6^ ** *p* < 0.05 TBI U46619 10^−6^ Ʃn= 24, *n* = 6 in each group).

Administration of 20-HETE (10^−7^ mol/L), known to be converted to the constrictor PGH_2_/TXA_2_ [28] and U46619, a stable analog PGH_2_, both acting on TP receptors elicited substantial and significant constrictions of MCA isolated from intact group of rats. (20-HETE 10^−7^ mol/L: −31 ± 3.6% U46619: (10^−7^ mol/L: −21 ± 3.3%, 10^−6^ mol/L: −29 ± 1.5%), whereas in MCAs from TBI rats, these responses were significantly reduced (20-HETE 10^−7^ mol/L: −16 ± 2.5%; U46619 −16 ± 2.4%; 10^−6^ mol/L: −16 ± 1.7%) (Figure 5).

## 4. Discussion

The novel findings of the present study are that traumatic brain injury (1) significantly reduces flow-induced constriction of middle cerebral arteries, (2) which corresponds to the reduction of arachidonic acid- 20-HETE- and thromboxane receptor agonist-induced constrictions. Thus, we propose that TBI interferes with the function of CYP450 4A, thereby with the production of constrictor prostaglandins and with the sensitivity of TP receptors to these prostaglandins, resulting in a substantially reduced flow-induced constrictor response. 

Based on the findings of present and previous studies [27], we propose that impairments of the pressure- and flow-induced constrictor mechanisms can importantly contribute to the dysfunction of the autoregulation of cerebral blood flow after traumatic brain injury.

### 4.1. Traumatic Brain Injury and Consequences

Every year, close to two million people in the USA suffer traumatic brain injury, whereas this number is about two and a half million in the European Union, not to mention other regions of the word. Young children and elderly people are especially exposed to this type of injury due to fall, accidents [35], or even violence. In addition, TBI occurs frequently due to the increasing popularity of sports and extreme sports and military actions [36]. There is a development in the treatment of TBI and its consequences, such as headache, micro-hemorrhages, and edema, but due to the still unknown pathological mechanisms, the success of treatments are limited. Because the brain is seated in the rigid cranium, regulation of intracranial volume and thus pressure is crucial to maintain healthy function of the brain [37]. We and others have shown that vasomotor mechanisms sensitive to hemodynamic forces (such as pressure and flow) are importantly involved in the maintenance of cerebral blood flow despite increasing systemic pressure i.e., autoregulation.

### 4.2. Traumatic Brain Injury Impairs Pressure and Flow Sensitive Vasomotor Mechanisms

Previously, we showed that vasomotor mechanisms sensitive to changes in hemodynamic forces are importantly involved in the autoregulation of cerebral blood flow [4,13,21]. The middle cerebral artery and its branches are in a key position to regulate the incoming blood volume to the brain. We found that the pressure sensitive constrictor mechanism of isolated rat middle cerebral arteries is severely impaired after traumatic brain injury [21]. Since we previously also showed that increases in flow also elicit constriction of MCA, thereby also contributing to the autoregulation of CBF [4,28,38,39], it was logical to hypothesize that this mechanosensitive response is also affected by TBI. We used pharmacological tools to gain insight into the molecular mechanisms mediating flow-induced vasomotor response and its changes after TBI.

Indeed, we found that the substantial flow-induced constriction of MCAs isolated from intact rats became severely mitigated after TBI (Figure 1). Similar reduction in flow-induced constriction was obtained when thromboxane-prostanoid A_2_ receptors (TP) were inhibited (with SQ 29,548) or when inhibitor of CYP450 4A (HET0016) was used, and the reduced responses were not further affected by TBI (Figure 1 and Figure 2). 

Next, we used AA, precursor of various prostanoids, to test the involvement of the AA-molecular pathway in TBI. In cerebral vessels, AA is metabolized by cytochrome P450 o-hydroxylases (CYP450 4A) into 20-HETE, and other prostanoids, which have important roles in the regulation of cerebrovascular tone [13,15,21,40]. Among others, these metabolites mediate agonists- and pressure-induced responses of cerebral vessels [18,19]. In the present study, we confirmed this by showing that AA, after an initial dilation, elicited constriction, thus from AA, via various enzymes (cyclooxygenases, CYPs, lipoxygenase) dilator and constrictor metabolites are produced [21,28]. Because TBI significantly reduced both dilator and constrictor responses to AA, we concluded that this pathway is impaired by TBI, which can primarily include the enzymes producing and/or receptors sensitive to constrictor prostanoids. 

We also found a substantial reduction in the expression of mRNAs of CYP450 subunits after TBI (Figure 4). Thus, on the basis of the present and previous findings that following TBI 20-HETE-mediated flow-induced constrictions of MCAs is substantially and significantly attenuated, we propose, that it is due to the decreased cerebrovascular production and impaired TP receptor mediated constrictor effect of 20-HETE. The first possibility is supported by the finding that after TBI, HET0016 inhibitor of CYP450 4A did not elicit further reduction in the constrictor response, and the expression of the 20-HETE producing CYP isoforms tended to decrease after TBI (albeit, the expression did not reach statistical significance due to large variation of data), whereas the second one is supported by the finding that constriction to U46619, a stable thromboxane analog acting via TP receptors became reduced after TBI. Certainly, cerebrovascular expression of CYP enzymes not necessarily correlates with the 20-HETE level produced in response to flow. This hypothesis therefore should be tested by direct measurement of the cerebrovascular 20-HETE level, as in previous studies [12,41].

In line with previous studies [10,42], we interpret these findings that, increasing in flow elicits the release of arachidonic acid (AA), which is then metabolized by CYP450 4A to 20-HETE, which is metabolized by COX into 20-endoperoxides (20-OH-PGH 2, 20-OH-PGG 2), causing constriction of arteries acting on TP receptors [4,21,28]. The findings that in a manner similar to the reduction of flow-induced constriction after TBI, HET0016 (inhibitor of CYP450 4A) reduced constrictions to AA, and that the CYP450 4A metabolite 20-HETE and to the TP receptor agonist U46619-induced constrictions were also reduced after TBI, suggest that both the production and sensitivity of these receptors are impaired by TBI. In other words, the findings of the present study suggest that flow-induced constrictor response of middle cerebral arteries is severely reduced by traumatic brain injury, likely due to the reduced ability of cytochrome P450 4A to convert arachidonic acid to constrictor prostaglandins and the mitigated sensitivity of TP receptors.

In addition to the above conclusion, one can hypothesize that TBI via altered 20-HETE production may affect BK_Ca_ channels (large-conductance Ca^2+-^activated K^+^ channel). Indeed, there are data in the literature showing that 20-HETE inhibits a BK_Ca_ [20,43]. Thus, one can imply that overactivation of BK_Ca_ channels, or less inhibition of BK_Ca_ channels by 20-HETE after TBI, may also explain the reduced constriction after TBI. However, it is difficult to make a final conclusion at present, because previous studies showed that inhibition of BK_Ca_ channels in MCA in the presence of high pressure did not significantly increase myogenic tone [44]. Whereas, after TBI, it was shown that the mRNA expression of these channels were upregulated and activated by H_2_O_2_ [27]. Thus, further studies are necessary to elucidate the potential role of BK_Ca_ channels in the reduced flow-induced constriction after TBI.

### 4.3. Consequences of the TBI-Impaired Mechanosensitive Vasomotor Mechanisms on the Autoregulation of CBF

**Figure 6:**
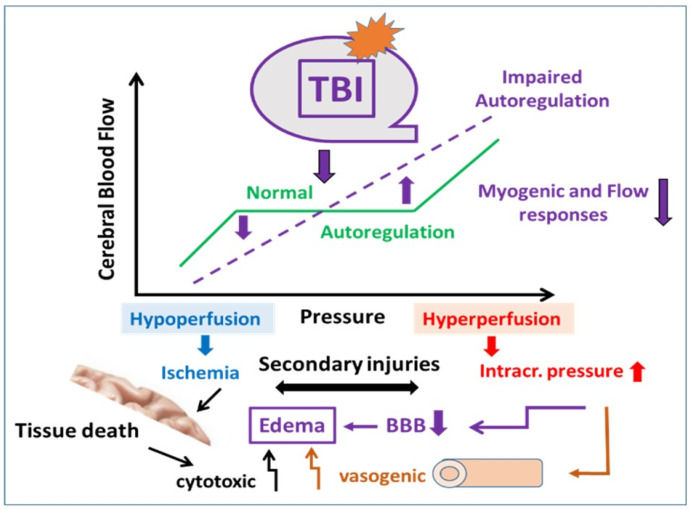
This figure shows the role of impaired pressure- and flow-sensitive mechanisms in the development of traumatic brain injury (TBI)-induced vasomotor dysfunction and brain edema. In normal, intact conditions, pressure- and flow-sensitive mechanisms maintain—in a wide range—a relatively constant cerebral blood flow despite of increasing perfusion pressure (autoregulation). After TBI, the impairment of these mechanisms develops, thus we propose that blood flow would increase as a function of pressure. In addition, in the lack of appropriate constrictions of larger and smaller arteries—not only CBF, intracranial volume and thus pressure increases as perfusion pressure increases—but also the distal microvessels—part of the blood brain barrier—become exposed to higher intraluminal pressure. These, together with the mediators released from the injured brain tissue, could lead to the opening of BBB, leakage of fluid, and various molecules, contributing to the development of edema and brain tissue injury.

Taken together previous and present findings and ideas, we developed a schema to better understand the effect of TBI on the autoregulation of cerebral blood flow and the secondary consequences (Figure 6). Figure 6 illustrates that in normal, intact conditions, pressure and flow sensitive constrictor mechanisms maintain—in a wide range—an idealized constant cerebral blood flow despite increases in perfusion pressure. In the absence or impairment of these mechanisms, such as after TBI, the diameter would not decrease substantially, thus-in extreme conditions-flow would increase linearly as a function of pressure. Since flow is related to the 4th power of the radius, a ~50 μm constriction of MCA to hemodynamic forces (pressure myogenic [27] and flow-induced, present study) can have significant effect on the amount of flow going through this vessel. In addition, in vivo flow elicits constrictions of not just a short segment of MCA, but the whole arterial and arteriolar network, which then can substantially limit the increase in blood flow. Impairments of such constriction of the whole vascular network (arterial and likely venular [33] after TBI can substantially increase flow and pressure, which can penetrate into the distal part of the brain microcirculation, leading to increases in blood volume and pressure, which are likely to be responsible for the development of edema and tissue toxicity (see Figure 6). High intra-capillary and post-capillary venular pressure, together with the mediators released from the injured brain tissue, can lead to the opening of the blood brain barrier (BBB), promoting the extravasation of fluid and various molecules, contributing to the development of edema and tissue injury (Figure 6). Impaired CBF autoregulation can lead to increased intracranial volume and thus intracranial pressure, severely affecting brain function, leading to mental disability, such as Alzheimer-type disease.

### 4.4. Limitation of the Present Study

One has to realize that, in the present study, we have used individual small isolated arterial vessels, in which it is difficult to perform molecular studies or measure the release of mediators before or after TBI. Changes in the level of AA metabolites in the cerebrovascular wall have been measured by direct measurement of cerebrovascular 20-HETE level, by liquid chromatography-mass spectrometry as in previous studies [12,41], but this is beyond the scope of the present paper, and unfortunately, such methods are not available for us at the moment. Lack of such measurement is a limitation to our studies. Additionally, regarding autoregulation, we assumed that other segments and branches of cerebral arteries behave the same manner after traumatic brain injury, and thus autoregulation. These later ideas, however, are supported by many clinical findings that, after head trauma, serious brain edema develops [45,46]. Additionally, it is certain that not only arterial but venular circulation is affected, together with damages of brain tissues during head trauma. All these limitation at the same time may assign further areas of research to alleviate the consequence of traumatic brain injury (concussion).

## 5. Conclusions

The present findings suggest that flow-induced constrictor response of middle cerebral arteries is severely reduced by traumatic brain injury, likely due to the reduced ability and expression of cytochrome P450 4A to convert arachidonic acid to constrictor prostaglandins and the mitigated sensitivity of thromboxane-prostanoid receptors. Taken together, the present and previous findings provide a mechanistic base for explaining the impairment of cerebral blood flow autoregulation and the consequent injury of the blood-brain barrier, development of microbleeds, edema, and mental dysfunction after brain trauma.

Thus, we propose that TBI interferes primarily with the function of CYP450 4A, thereby with the production of constrictor prostaglandins and with the sensitivity of TP receptors to these prostaglandins, resulting in a substantially reduced flow-induced constrictor response. Based on the findings of present and previous studies [32], we propose that impairments of the pressure- and flow-induced constrictor mechanisms can importantly contribute to the dysfunction of the autoregulation of cerebral blood flow after traumatic brain injury. Addressing these mechanisms by newly developed pharmacological therapeutics may lead to alleviation of cerebrovascular and brain damages induced by traumatic brain injury. 

## Data Availability

Data available from the first author (ancsi.szenasi@gmail.com).
